# Hereditary Hemochromatosis: A Cardiac Perspective

**DOI:** 10.7759/cureus.20009

**Published:** 2021-11-29

**Authors:** Pranay K Joshi, Saawan C Patel, Devarashetty Shreya, Diana I Zamora, Gautami S Patel, Idan Grossmann, Kevin Rodriguez, Mridul Soni, Ibrahim Sange

**Affiliations:** 1 Internal Medicine, B.J. Medical College, Ahmedabad, IND; 2 Internal Medicine, Pramukhswami Medical College, Karamsad, IND; 3 Internal Medicine, Gandhi Medical College, Secunderabad, Hyderabad, IND; 4 General Medicine, Universidad de Ciencias Médicas Andrés Vesalio Guzman, San José, CRI; 5 Research, Medical University of Silesia, Katowice Faculty of Medical Sciences, Katowice, POL; 6 Research, Universidad Americana Managua (UAM), Managua, NIC; 7 Pediatric Gastroenterology, Shri Lal Bahadur Shastri Government Medical College and Hospital, Mandi, IND; 8 Research, K. J. Somaiya Medical College, Mumbai, IND

**Keywords:** cardiac arrhythmia, primary iron overload, cardiac hemochromatosis, iron overload cardiomyopathy, hereditary hemochromatosis

## Abstract

Hereditary hemochromatosis (HH) is a common genetic metabolic disorder characterized by excessive iron absorption and elevated serum iron levels, which accumulate in various organs, such as the heart, pancreas, gonads, and damage these organs. There are only a few articles and clinical studies describing the characteristics of cardiac involvement in HH along with the significance of early diagnosis and management in preventing complications. In this review article, we have reviewed multiple pieces of literature and gathered available information regarding the subject. We compiled the data to investigate the importance of early detection of symptoms, regular monitoring, and prompt management with strict adherence to reverse or prevent complications. This article has reviewed different aspects of cardiac hemochromatosis, such as pathogenesis, clinical presentation, diagnosis, and management. Recognition of early symptoms, diagnosis of cardiac involvement with various modalities, and implementation of early treatment are essentially the foundation of better outcomes in HH.

## Introduction and background

HH is an inherited, multi-systemic, progressive metabolic disease characterized by systemic iron overload and iron deposition in various visceral organs [1]. It is one of the most common inherited metabolic diseases worldwide and predominantly found among the Caucasian population with a frequency of the mutated gene C282Y found in one to two individuals in every 500 and less frequently found among Hispanics, Asians, and African Americans [2].

Historically, the first case of HH was described by French physician Armand Trousseau in the mid-19th century [3]. In 1935, Joseph Sheldon, a British geriatrician, described 311 case records in his monologue and concluded that hemochromatosis is an inherited disorder and resulted from the accumulation of iron in multiple organs [4]. Hemochromatosis was first linked to the HFE gene in 1996 by Feder et al.; 83% of patients were homozygous for missense mutation of HFE in the study [5]. Later on, multiple other genes were identified responsible for a minority of cases of HH such as hemojuvelin (HJV), transferrin receptor 2 (TfR2), and ferroportin [6-8]. The genetic mutations that contribute to the pathogenesis of HH are multiple and have an expected outcome of excessive iron concentration in plasma.

The pathogenesis of HH involves the failure of the downregulation of iron metabolism. HFE-related hemochromatosis results from abnormally low levels of hepcidin, a key regulator of iron absorption from the duodenum, and the release of iron from the macrophage, secreted by the hepatocytes in response to decreasing plasma levels of iron [9]. The disorder can present with various symptoms, the most common being fatigue, malaise, and arthralgia [10]. In any individual with suspicion of HH, measurement of serum iron, ferritin, and transferrin saturation (TS) is recommended [11]. Management primarily involves the reduction of serum iron as well as total body iron by phlebotomy, as it has shown to slow the course of the disease, reverse organ damage, and prevent long-term complications [11-12]. Iron chelators are alternative therapy required in patients with anemia requiring chronic blood transfusion, unstable hemodynamic status, and a few refractory cases [13-14]. Heart failure and hepatic carcinoma are leading causes of mortality in HH, and approximately one-third of patients succumb to death as a result of cardiac causes [15-16]. The cardiac involvement in HH can have an insidious progression that eventually, over the course of the disease, leads to irreversible myocyte damage as the cells are overwhelmed by the deposition of iron, making the management of such cases challenging [17].

For reasons mentioned in the preceding paragraph, it is of paramount importance to detect cardiac involvement in patients of HH at the earliest, with prompt initiation of treatment before the advent of complications.

In this article, we discuss the following aims and objectives: 1. Explore the early manifestations of cardiac hemochromatosis; 2. Emphasize the importance of early diagnosis of cardiac involvement; 3. Outline various modalities of management of cardiac hemochromatosis.

## Review

Iron metabolism and pathogenesis of cardiac hemochromatosis

Iron has a substantial role in various biochemical reactions, and it is also the main component of biomolecules, such as hemoglobin and myoglobin, and multiple enzymes [18]. Furthermore, iron catalyzes oxidative reactions, which generate reactive oxygen species (ROS) [19-20]. Hence, excessive amounts of iron will lead to grave effects by generating excessive amounts of ROS, which mandates strict control of its level in the body. There is no specific pathway of iron excretion from the body except the losses that occur through the shedding of intestinal mucosal cells and menstruation in females [21]. Therefore, the most effective way to control the iron level in the body is through restricted absorption and not excretion.

**Figure 1:**
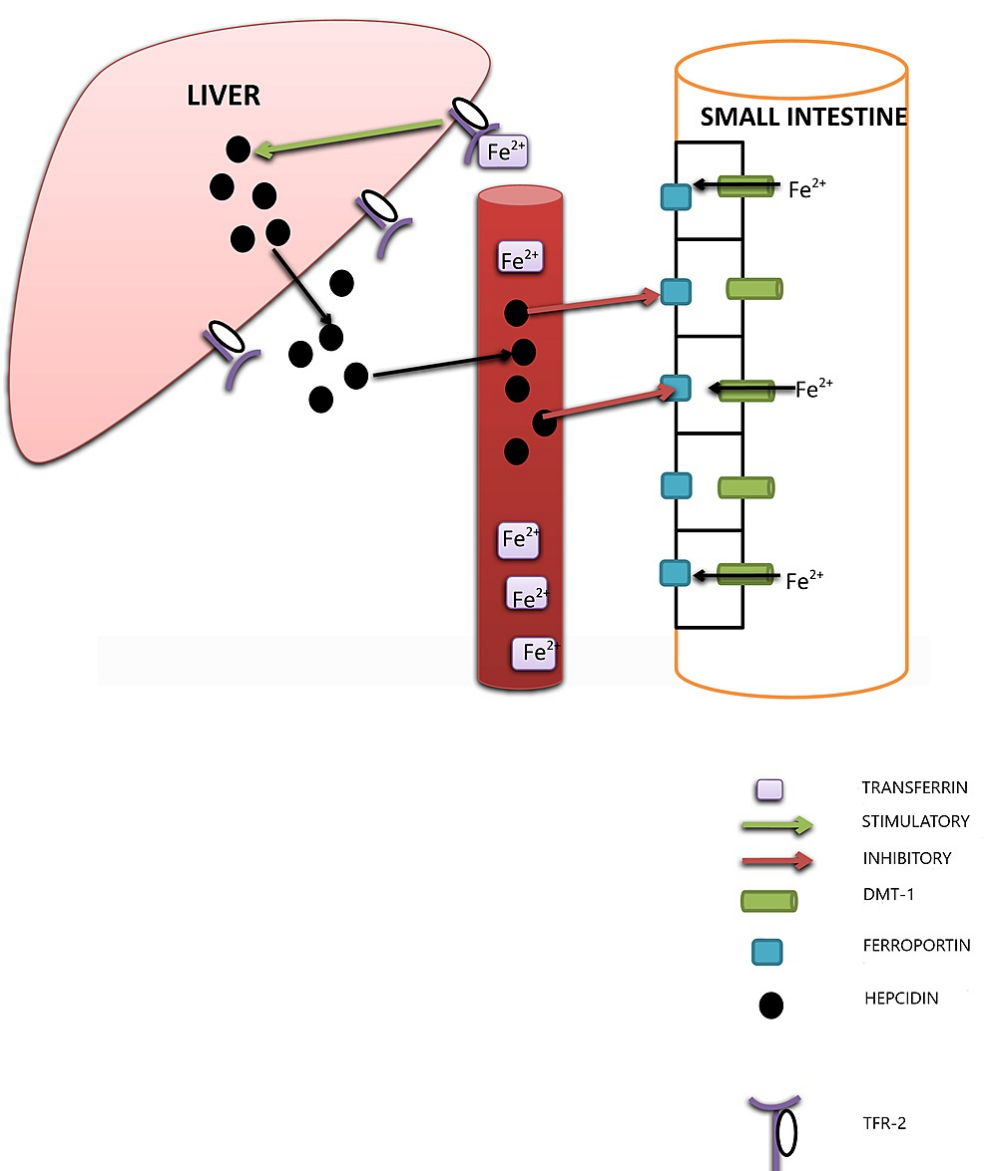
Schematic representation of iron metabolism and regulation DMT-1: divalent metal transporter-1; TFR-2: transferrin receptor-2

Hepcidin controls the activity of ferroportin, an iron exporter present on the basolateral surface of enterocytes, macrophages, and hepatocytes (Figure 1) [22]. Although mechanisms and mediators responsible are different, all types of HH, in common, have abnormally low hepcidin levels. Reduced hepcidin level manifests as uninhibited activity of ferroportin and resultant excessive iron absorption, especially in classical HH [23]. More importantly, in classical HH, the macrophage plays a significant role than the duodenal enterocytes [23]. In patients of HH, macrophages release more iron than an average individual [24]. Irrespective of the clinical subtype, HH invariably involves dysfunctional iron metabolism. Usually, the body absorbs 1 to 2 mg iron daily; in HH, however, this reaches up to 8 to 10 mg per day [25]. Initially, transferrin saturation increases, which is the earliest biochemical marker of iron overload [26]. Gradually, as transferrin saturation reaches 75%, further iron remains in free form in the plasma, which has been termed non-transferrin-bound-iron (NTBI) and is central to the pathogenesis of HH, mainly through the generation of reactive oxygen species [19].

HH can be divided into various subtypes (Table 1) [27].

**Table 1:** Clinical subtypes of hereditary hemochromatosis HAMP: hepcidin anti-microbial peptide; HJV: hemojuvelin; tfr-2: transferrin receptor-2

SUBTYPE	INHERITANCE	MUTATED GENE	SEVERITY	AGE OF ONSET	AFFECTED POPULATION	PHENOTYPE
Type 1	Autosomal Recessive	HFE	Mild	4-5 decade	Middle-aged white male	Liver disease, Arthralgia, Fatigue
Type 2	Autosomal Recessive	2a: HJV	Severe	1-2 decade	Young, white/non-white, Male/female	Cirrhosis, Arrhythmia, Heart failure
		2B: HAMP				
Type 3	Autosomal Recessive	tfr-2	Mild to severe	3-4 decade	Young, white/non-white, Male/female	Endocrinopathy, Cardiomyopathy, Liver failure
Type 4	Autosomal Dominant	Ferroportin	Mild	4-5 decade	White/non-white, Male/female of all ages	Liver abnormalities, Marginal anemia, Hyperferritinemia

Cardiac hemochromatosis is a common occurrence in patients of HH. It involves a multitude of presentations, which include but are not limited to cardiomyopathy, arrhythmia, and sudden cardiac death [28]. Gradually, in HH, as iron concentration builds up, the free iron eventually starts to accumulate in the heart. The free iron enters cardiomyocytes through the L-type calcium channel (LTCC) [29]. The free iron generates ROS through the Fenton reaction. It dramatically accelerates the production of hydroxyl ions, which damage the organelle membrane, cell membrane, and lysosomes, eventually leading to cell death in the heart [30]. Since cardiac myocytes have a high number of mitochondria and fewer antioxidants, they are particularly susceptible to damage from ROS [31]. All the subtypes have similar syndromic presentations because of identical pathogenesis and organ involvement. The only difference is the severity of the disease, with the juvenile form of HH involving the heart at an early stage with rapid deterioration [28]. Excess iron deposition begins from the epicardium and progresses towards the endocardium, which explains the preservation of ventricular systolic function till late in the disease [32]. In contrast to the previously held opinion that free iron gets deposited in the interstitial space, recent histological observations have shown that the iron is indeed deposited in the sarcoplasm [33]. Therefore, iron deposition in the heart is a form of storage rather than an infiltrative process, and potentially, it can be reversed with the initiation of appropriate therapy.

Cardiac hemochromatosis: clinical presentation

About 15% of HH patients have cardiac symptoms as a chief complaint. Symptoms appear insidiously and progress gradually, consisting of non-specific symptoms such as malaise, fatigue, and lethargy that worsen over time [28]. These symptoms are mainly the result of cardiomyopathy that can be either restrictive or dilated and has a progressive course that can result in death due to congestive heart failure if not treated promptly. Palpitations and sudden cardiac death occur due to iron deposition in the cardiac conduction system [34]. Udani et al. conducted a retrospective cohort study on 63,846,188 hospitalized patients to determine the prevalence and risk of CV manifestations in patients with hemochromatosis and discovered that among 64,590 patients with hemochromatosis, 27.8% had one or more cardiovascular manifestations [35]. Among CV manifestations, arrhythmias, congestive heart failure (CHF), and pulmonary hypertension were the most common [35].

**Figure 2:**
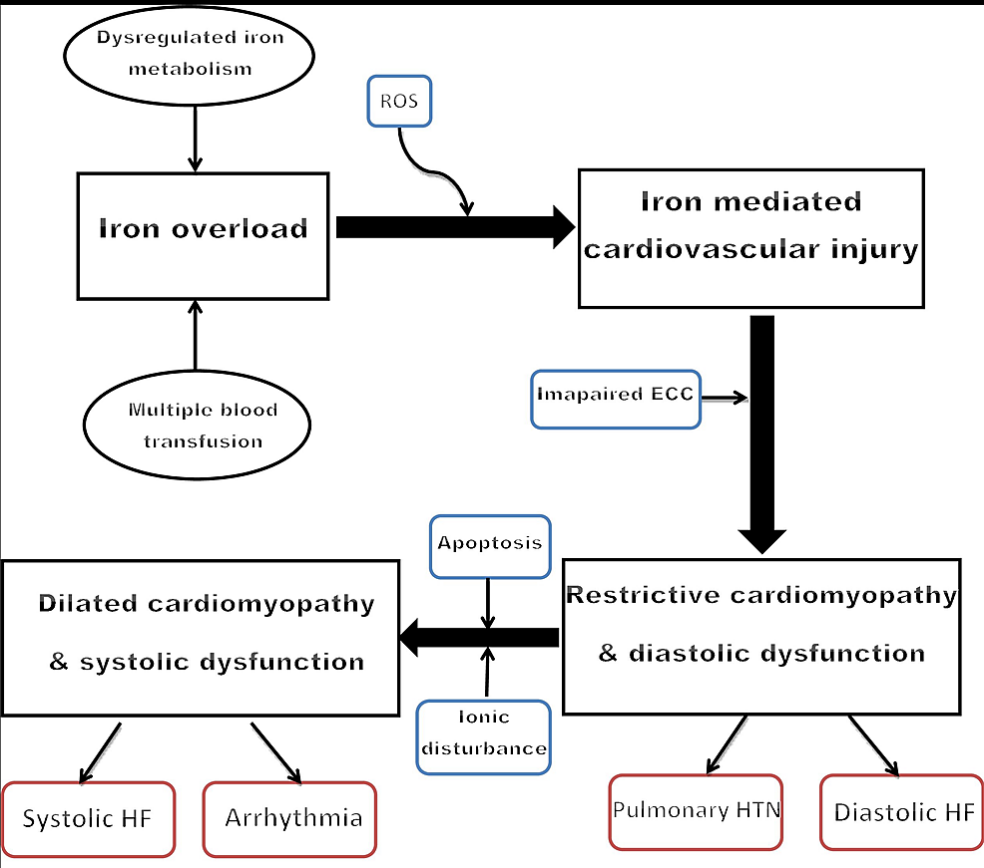
The spectrum of iron overload cardiomyopathy ROS: reactive oxygen species; ECC: excitation-contraction coupling; HF: heart failure; HTN: hypertension

Iron overload cardiomyopathy (IOC) can be either systolic or diastolic cardiac dysfunction originating from iron accumulation in cardiomyocytes, independent of other concomitant abnormalities such as ischemic cardiomyopathy, atherosclerosis, or valvular diseases [36]. IOC is a cardinal cause of mortality, especially in the second and third decades of life. In the long term, it has been called one of the most significant predictors of mortality in patients with iron overload [37]. IOC has common characteristics and progresses in a predictable way irrespective of the type of hemochromatosis, characterized by early restrictive cardiomyopathy (RCM) with diastolic dysfunction, which, if not treated in the early stage, invariably progresses to late dilated cardiomyopathy (DCM) with impaired systolic function (Figure 2) [38]. Oxidative stress in cardiomyocytes due to excessive free iron accumulation leads to impaired excitation-contraction coupling (ECC) and impaired function of sarcoendoplasmic reticulum calcium ATPase 2 (SERCA2) and sodium-calcium exchanger (NCX) channel leading to high intracellular calcium and sodium concentration, respectively [39-40]. These ionic changes within cardiomyocytes collectively lead to reduced peak systolic and high diastolic calcium levels, resulting in impaired systolic and diastolic function, respectively [41]. After entering into cardiomyocytes through L-type calcium channels, Fe+2 leads to the slowing of the Ca+2 current resulting in high intracellular Ca+2, which explains the early diastolic dysfunction [29]. Later on, with persistently elevated levels of Fe+2, transport of Ca+2 across the membrane is impaired because Fe+2 competes for the same iron transporter, which manifests as late systolic dysfunction seen in IOC [29]. Initially, patients have no overt symptoms but eventually, as diastolic dysfunction develops, non-specific symptoms such as mild to moderate exertional fatigue and exercise intolerance, appear [42]. Shizukuda et al. carried out a study on 43 asymptomatic HH patients with echocardiographic monitoring to evaluate the relationship between left ventricular diastolic function and oxidative stress and demonstrated a significant association between oxidative stress and left ventricular diastolic dysfunction in patients with HH in the early stage, with no overt symptoms and more than 55% ejection fraction (EF) [43]. Early in the disease, restrictive cardiomyopathy presents as diastolic heart failure with symptoms such as exertional dyspnea, exercise intolerance, orthopnea, peripheral swelling, and paroxysmal nocturnal dyspnea [44]. Later, untreated cases progress to irreversible dilated cardiomyopathy due to apoptosis of cardiomyocytes because of long-term iron deposition and subsequent damage by ROS. Dilated cardiomyopathy presents as systolic dysfunction and is often biventricular.

Rhythm abnormalities in cardiac hemochromatosis are common. Its etiopathogenesis involves iron deposition in the sinoatrial (SA) node, atrioventricular (AV) node, and cardiac conduction system [45]. In a clinical study undertaken by Shizukuda et al., 42 asymptomatic patients with homozygosity of C282Y showed no significant incidence of arrhythmia. Over six months, reducing iron levels in asymptomatic HH patients did not statistically reduce the incidence of arrhythmias [46]. They concluded that oxidative stress, rather than systemic iron-overload, is the main culprit behind arrhythmia in HH. After treatment with multiple phlebotomies, subjects have constantly elevated oxidative stress, which explains the persistence of arrhythmia in patients with near-normal serum iron levels [46]. These abnormalities include atrial tachyarrhythmias, premature ventricular beats, nodal block, and ventricular tachycardia, among which supraventricular arrhythmia is the most common cardiac rhythm disorder [16,47]. The iron itself is arrhythmogenic, as it can interfere with the cardiac conduction system [48]. Kenneth et al. conducted a study on the experimental animal model of IOC to determine the molecular and cellular mechanisms responsible for arrhythmia in subjects with iron overload. They hypothesized and demonstrated that iron-induced reduction in the sodium current is central to the pathogenesis of arrhythmia [49]; apart from this, they discovered fixed regions of conduction block that contribute to arrhythmia by establishing a re-entrant circuit [49]. Thus, action potential shortening and abnormal electrical impulse conduction due to heterogeneous iron deposition are essentially responsible for unidirectional block and arrhythmia in hemochromatosis [50]. Irregular rhythm may be due to atrial fibrillation, premature atrial contraction, or premature ventricular contractions. On the other hand, regular rhythm with rapid heart rate is due to atrial flutter, paroxysmal supraventricular tachycardia, or sustained ventricular tachycardia [50]. Symptoms may include palpitation, fatigue, chest discomfort, and frank syncope resulting from bradyarrhythmias [51].

There is little evidence to support the exact pathophysiological mechanism accounting for pulmonary hypertension in hemochromatosis. It is theorized that iron-induced damage of endothelium results in vasoconstriction in addition to smooth muscle proliferation of the media [52]. Sudden cardiac death that occurs in hemochromatosis is due to end-stage irreversible cardiomyopathy culminating in cardiogenic shock or fatal arrhythmia [35].

Diagnosis

With the advancement in diagnostic techniques, numerous patients with cardiac involvement in HH are diagnosed early. Still, a large number of patients remain undiagnosed or underdiagnosed. It is imperative to detect cardiac disease in the early asymptomatic stage before it progresses to irreversible cardiomyopathy and subsequent heart failure. Early diagnosis though challenging, is essential, and various modalities, such as biochemical, genetic, radiological, and histological analysis, should be incorporated [53]. Physical examination and thorough history evaluation are similarly essential. A significant degree of cardiac dysfunction has already occurred before symptoms of cardiac hemochromatosis appear. Hence, early detection using sensitive markers is vital.

Serum transferrin saturation more than 55% and ferritin levels greater than 200 in pre-menopausal women and 300 in men and post-menopausal women is called iron overload [54]. Both markers, however, have low sensitivity and specificity. Their levels are also elevated in inflammatory and infectious conditions. Conversely, high cardiac iron accumulation can also occur in the setting of low levels of serum ferritin, suggesting no significant correlation between serum ferritin levels and severity of myocardial involvement [55]. These markers are chiefly helpful for screening purposes. Genetic screening for C282Y mutation is widely available. It helps to detect family members who are homozygous for the mutation.

Transthoracic echocardiography (TTE) is an inexpensive, non-invasive, and widely available technique to screen patients for cardiac hemochromatosis. It is challenging to diagnose early diastolic dysfunction by using M-mode echocardiography. Changes observed are increased left ventricle (LV) early diastolic filling and left atrial (LA) dimensions with preserved systolic function [56]. Enhanced left atrial contractile function that occurs even before LV diastolic dysfunction may be the earliest marker of cardiac iron overload on echocardiography [57]. Tissue Doppler imaging (TDI) is a modern technique to diagnose early involvement of the heart in HH. Palka et al. tested a hypothesis in their study on 18 HH patients that LV diastolic filling indexes are helpful in early detection of cardiac involvement in HH and used TDI as a diagnostic imaging technique. They observed a reduction in peak systolic and early diastolic filling using TDI and concluded that TDI is a valuable tool for early detection of cardiac hemochromatosis [58].

Magnetic resonance imaging (MRI) is the best non-invasive method to detect iron-overload quantitatively in the heart. Iron in tissues acts as a paramagnetic force and disrupts the magnetic signals received by the detector, which results in a heterogeneous image of the tissue displayed as early darkening on the MRI [59]. Cardiac MRI using late gadolinium enhancement (LGE) is a useful technique to identify the extent of cardiac tissue fibrosis that transcends non-contrast MRI for comprehensive cardiac assessment. The sensor in MRI refocuses on the radio waves derived from the tissue, which is called echo time. In tissues with iron overload, as the echo time increases, the darkening of images occurs earlier. The time constant for gradient echo is called T2*. T2* is a sensitive and highly specific method that can quantify and track myocardial iron overload [60]. Mid-ventricular iron overload is representative of global cardiac iron overload and can quantitate cardiac iron deposition [60]. Furthermore, MRI can also be used to determine ejection fraction, cardiac tissue mass, and viability.

Management

Therapeutic phlebotomy and iron-chelators are the mainstays of therapy. Early initiation of treatment can reverse LV dysfunction, and as the iron deposition is not interstitial, cardiac function can be restored entirely [33].

Phlebotomy is the least toxic and most effective method to control serum iron levels. It entails the removal of 400 to 500 milliliters of blood, which roughly correlates to 200 to 250 mg of iron at each session once or twice, initially. Around 1 to 2 gm iron can be removed per month [61]. This treatment helps to mobilize iron from tissues such as the heart, liver, and pancreas. The target of the therapy is to achieve serum ferritin levels below 30 ng/ml and transferrin saturation less than 30% [62]. After the target level is reached, maintenance treatment every two to three months is required with strict monitoring of hematocrit and serum iron levels. This frequency varies among patients depending on the iron reaccumulation rates [63]. A study on 22 patients with HH carried out by Candell-Riera et al. to determine the efficacy of phlebotomy for management of HH demonstrated significant improvement in LV function after a long-term treatment [56]. Echocardiographic findings showed a reduction in diameter and mass of LV in patients with abnormal LV parameters initially [56].

Iron-chelators are used in refractory cases and patients with CHF. They increase the rate of iron excretion by binding iron in plasma and tissue [64]. Deferoxamine, deferiprone, and deferasirox have been approved as iron-chelators. Deferoxamine is given subcutaneously or intravenously while the other two are delivered through the oral route. Intensive combination therapy with deferoxamine and deferiprone rapidly improves cardiac function to the normal state and reduces serum iron levels [65]. A new therapeutic approach includes calcium channel blockers (CCB). CCBs block iron uptake of cardiomyocytes through LTCC and prevent deposition of iron in the heart. An experimental study on mice demonstrated that amlodipine reduces iron uptake in the heart and reduces the amount of ROS generation [66]. It is imperative to initiate guideline-directed medical treatment (GDMT) for CHF, such as angiotensin-converting enzyme inhibitors (ACEI), angiotensin receptor blockers (ARB), diuretics, beta-blockers, and inotropes, in patients who develop heart failure in the late stage of the disease. ACEI and ARB prevent remodeling of the heart and worsening of CHF. Beta-blockers decrease myocardial oxygen consumption and reduce mortality in the long term.

Limitations

This article solely focuses on cardiac abnormalities arising due to iron deposition in the heart and ignores other co-existing factors that can contribute to the modification of cardiac functions. Practically, many factors can influence cardiac functions, including environment, comorbidities, and genetic predisposition. The article has not considered disparities in the genetic structure of individuals that can result in certain people developing more severe and rapid cardiac dysfunction.

## Conclusions

It is evident from the review article that, relatively, there are few studies regarding the emphasis on the early recognition and treatment of cardiac hemochromatosis. To summarize, HH can present as iron overload cardiomyopathy, arrhythmias, pulmonary hypertension, and CHF. IOC evolves predictably, beginning as RCM with diastolic dysfunction and, if left untreated, progresses to DCM with systolic dysfunction. CHF ensues as IOC progresses. Iron deposition in the heart generates ROS, damaging cardiomyocytes and triggering a cascade leading to impaired cardiac function. Diagnostic approaches to detect IOC are echocardiography, Doppler imaging, and cardiac MRI. Family members of patients should be screened for HH by genetic techniques. Cardiac function should be regularly monitored in asymptomatic patients and patients with symptoms other than cardiac. Cardiac hemochromatosis is best managed by therapeutic phlebotomy, and refractory cases are treated with iron chelators. The clinical implication of this article is to give a glance at various aspects of cardiac hemochromatosis. This article can serve as a shared resource to obtain knowledge, gain deep insight, and guide physicians to acquire additional information regarding the topic. Lastly, we feel that active investigation through clinical studies and systemic analysis is required to form a rigid construct and manage the condition more efficiently.
